# Exosomes, MDSCs and Tregs: A new frontier for GVHD prevention and treatment

**DOI:** 10.3389/fimmu.2023.1143381

**Published:** 2023-03-29

**Authors:** Nicholas J. Hess, John A. Kink, Peiman Hematti

**Affiliations:** ^1^ Division of Hematology, Oncology and Palliative Care, Department of Medicine, University of Wisconsin School of Medicine and Public Health, Madison, WI, United States; ^2^ University of Wisconsin Carbone Cancer Center, Madison, WI, United States

**Keywords:** graft vs host disease, exosomes, regulatory T cells, myeloid derived suppressor cell (MDSC), mesenchymal stromal cell (MSC)

## Abstract

The development of graft versus host disease (GVHD) represents a long-standing complication of allogeneic hematopoietic cell transplantation (allo-HCT). Different approaches have been used to control the development of GVHD with most relying on variations of chemotherapy drugs to eliminate allo-reactive T cells. While these approaches have proven effective, it is generally accepted that safer, and less toxic GVHD prophylaxis drugs are required to reduce the health burden placed on allo-HCT recipients. In this review, we will summarize the emerging concepts revolving around three biologic-based therapies for GVHD using T regulatory cells (Tregs), myeloid-derived-suppressor-cells (MDSCs) and mesenchymal stromal cell (MSC) exosomes. This review will highlight how each specific modality is unique in its mechanism of action, but also share a common theme in their ability to preferentially activate and expand Treg populations *in vivo*. As these three GVHD prevention/treatment modalities continue their path toward clinical application, it is imperative the field understand both the biological advantages and disadvantages of each approach.

## Introduction

Graft versus-host disease (GVHD) remains a significant problem following an allogeneic hematopoietic cell transplantation (allo-HCT) with GVHD patients experiencing a lower quality of life and a higher risk of death. Our understanding of the cellular and molecular mechanisms of GVHD has provided researchers additional clarity on the etiology of its pathogenesis. This has allowed for a growing number of non-biologic and biologic-based GVHD prophylaxis/treatment therapeutics to be investigated as safer modalities for allo-HCT patients. Specifically, the investigation into the use of biologics for GVHD therapies is still in its infancy but there are several exciting possibilities in the pipeline. In this mini-review, we will highlight three emerging biologic-based therapies that are at different stages of conceptualization and development which include T regulatory cells (Tregs), myeloid-derived-suppressor cells (MDSCs) and mesenchymal stromal cell (MSC) exosomes. We will also emphasize the similarities and differences of each modality in terms of biologic activity and possible clinical use.

## Tregs as a GVHD prevention/treatment modality

**Figure 1:**
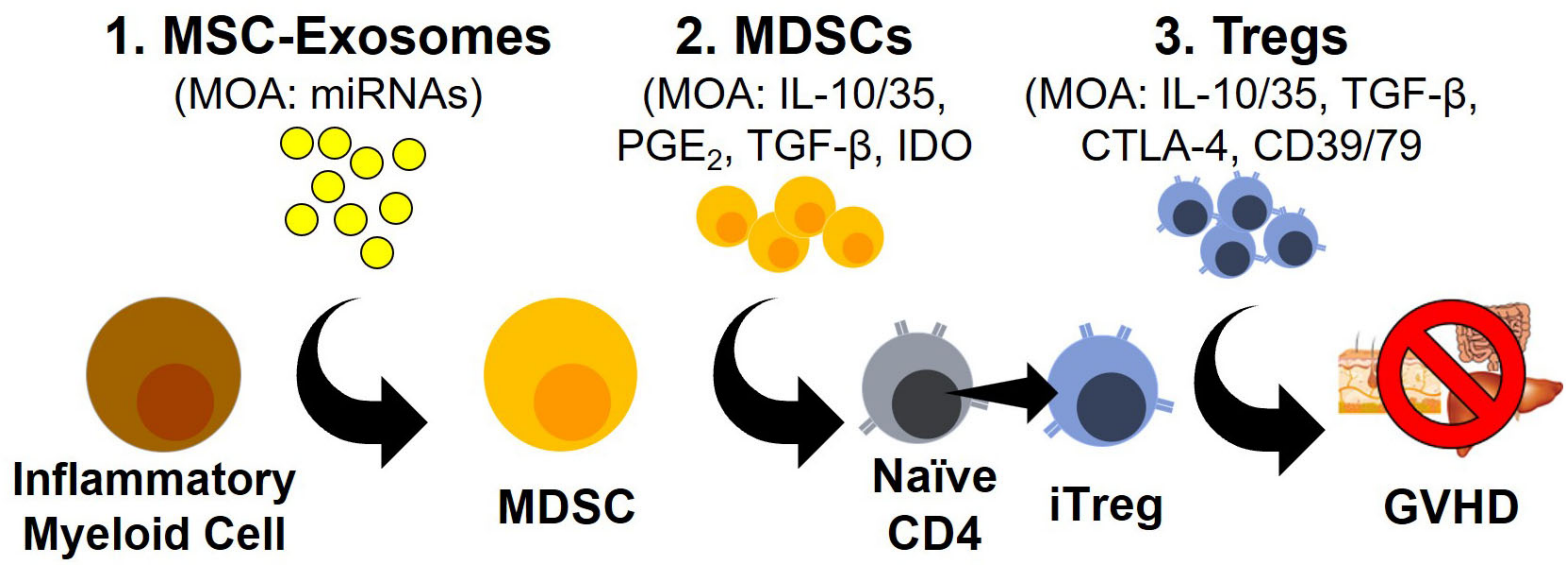
Novel Modalities For GVHD Prophylaxis/Treatment. Schematic outlining three emerging approaches to GVHD prophylaxis and treatment with their corresponding mode of actions (MOA).

T regulatory cells (Tregs) represent a T cell polarization state with potent immunosuppressive activity (1–4). Classically restricted to the CD4 lineage, an analogous T cell population within the CD8 lineage has now also been described (5, 6). Irrespective of the lineage, Tregs are best characterized by the transcription factor FOXP3 as well as high levels of the IL-2Rα subunit (CD25) at baseline (3). Upon activation, Tregs dampen immune responses through the secretion of the immunosuppressive cytokines IL-10, IL-35 and TGF-β, by modifying the expression of the inhibitory ligands CTLA-4 and by decreasing the inflammatory environment through the consumption of IL-2 and eliminating extracellular ATP *via* CD39/CD73 activity (3, 7) (**Figure 1**).

While the activity of Tregs is predicated on the recognition of self-peptides presented by antigen-presenting-cells (APCs) to prevent the development of an autoimmune disease, Tregs can also develop against non-self-peptides in the periphery (8). Moreover, there are two sub-types of Tregs, thymus-derived Tregs (tTregs) and induced-Tregs (iTregs) that each have non-overlapping roles in controlling immune responses (7, 9).

### Differences between induced and thymic Tregs during allo-HCT

Classical or thymus-derived Tregs (tTregs) develop during T cell development and are characterized in mice by the expression of neuropilin-1, the transcription factor Helios and in the expression of CD3, CD4, FOXP3 and CD25^high^ characteristic of all Treg populations (10–13). Most T cell clones that develop TCRs with high affinity toward self-peptides in the thymus are eliminated but a subset develop into tTregs that egress into the periphery (8). This Treg subset is generally thought to be terminally differentiated and act as a separate checkpoint to ensure immune responses are kept under control during steady state conditions (4).

In contrast, induced-Tregs (iTregs) exit the thymus as conventional T cells but are polarized to iTregs in environments with high levels of TGFβ, retinoic acid, IDO, and other mediators (8, 14, 15). The function of iTregs is in controlling immune responses against the commensal microbiota and in mucosal tolerance (16, 17). The pathway controlling the polarization of iTregs is similar to TH_17_ polarization. Studies have shown that iTregs can be polarized into TH_17_ cells and vice versa, highlighting the plasticity of this population and how the balance of iTreg/TH_17_ polarization can influence immune responses (18–20).

In both pre-clinical mouse models and clinical studies, it has been established that elevated Tregs frequencies in the graft is correlated with lower rates of GVHD (21–26). Another recent study using xenogeneic transplantation found elevated levels of Tregs early after transplant was predictive of survival (27). This study also translated their findings into the clinic and revealed that high levels of Tregs between days 7-17 post-transplant was associated with a GVHD-free, relapse-free outcome (27). However, whether the Tregs generated in this xenogeneic system or in patients represents the expansion tTregs or the polarization of iTregs remains unanswered.

The TCR repertoire of tTregs and iTregs do not overlap, highlighting the different antigens they recognize (germline self-antigens vs microbiota-derived antigens) (9, 28, 29). One requirement of tTreg antigen recognition is the correct self-antigen peptide sequence presented by the same HLA molecule it was initially derived from in the thymus. During an allo-HCT, where the HLA repertoire may be mismatched, this recognition process may be subverted. While HLA mismatched transplants are thought to have greater alloreactivity due to the larger proportion of TCR clones that can recognize allo-antigens, the lack of recognition between tTregs and their cognate antigens may also play a factor (30, 31).

Currently, there are no conclusive studies that have investigated the development of tTregs versus iTregs after allo-HCT. Ongoing studies differ based on their isolation and expansion of tTregs from existing graft material and those that polarize CD4 T cells *in vitro* (32). In addition to the origin of Tregs, ongoing studies will have to address the HLA disparity between donor and recipient in regard to their efficacy.

### Generating iTregs for allo-HCT

Many studies have highlighted the role of metabolism in the development of iTregs. While TGFβ is classically required for the generation of iTregs, the mammalian target of rapamycin (mTOR) may be equally important (33–35). mTOR contains two complexes, mTORC1 and mTORC2, which are both serine/threonine protein kinases that integrate signals from upstream nutrient sensing receptors. A knockout of both mTOR complexes in mice results in naïve CD4+ T cells only capable of polarizing to iTregs while the knockout of mTORC1 prevented TH_17_ polarization (36, 37). Additional studies have shown that an activated mTOR complex promotes the expression of HIF-1α that drives TH_17_ polarization (35). Conversely, blocking mTOR signaling with rapamycin (also known as sirolimus), skews CD4 T cell polarization to iTregs over TH_17_ cells (33–37).

The polarization of naïve CD4 T cells to iTregs by sirolimus makes it an effective therapeutic candidate that is currently used in the clinic. One multicenter study (BMT-CTN-1501) revealed that sirolimus was equivalent to prednisone for initial GVHD treatment and resulted in a shorter time to immune suppression discontinuation (38). Another phase 2 study revealed that the addition of sirolimus to cyclosporine and mycophenolate mofetil GVHD prophylaxis reduces the incidence of GVHD after nonmyeloablative HLA-mismatched transplantations compared to historical controls (39). However, the promise of sirolimus-based prophylaxis has been overshadowed slightly by the rapid utilization by the transplant community of post-transplant cyclophosphamide (PTCy), a modality that has also been shown to promote Treg expansion (40–42). Since both sirolimus and calcineurin inhibitors (e.g. tacrolimus and cyclosporine) bind to FKBP12 before their target of interest (mTOR and calcineurin respectively), sirolimus has most recently been studied as a replacement for tacrolimus in GVHD prophylaxis regimens with two studies showing favorable results when used with PTCy for haploidentical and mismatched transplants (43, 44). Belumosudil, a ROCK2 inhibitor approved for chronic GVHD, is another GVHD treatment drug that preferentially targets iTregs. Belumosudil promotes STAT5 versus STAT3 signaling to expand and active iTregs (45–47).

With so many current GVHD drugs connected with an expansion of the iTreg population, it is logical for a growing number of clinical trials to investigate the direct treatment of Tregs. Unfortunately, iTreg adoptive therapies are hindered by high production costs, the inability to generate/store multiple doses and the need for a patient specific product. Thus, the investigation into new therapies preferentially expand/polarize iTregs *in vivo* should continue to be prioritized.

## The role of MDSCs in controlling GVHD

Myeloid-derived-suppressor-cells (MDSCs) represent a heterogeneous population of cell types with the general function of dampening immune responses (48–50). MDSC were first viewed as a subset of immature myeloid cells that failed to differentiate properly during myelopoiesis due to the inflammatory environment caused by malignant cells. Recent evidence suggests that MDSC are also composed of mature myeloid cells that have transitioned/polarized into MDSCs. As such, of MDSC have been characterized into three main groups; immature-MDSCs (i-MDSC), granulocytic-MDSCs (G-MDSC) and monocytic-MDSCs (M-MDSCs) (48–51).

While markers for each MDSC population have been established in mice, a standardized set of human markers has not yet been widely accepted. Generally, human M-MDSCs share similar forward and side scatter characteristics as monocytes, are lineage positive for monocytes (CD14^+^, CD33^high^) but also express several M2 markers (CD124^+^, CD163^+^, CD206^+^) and are CD16^-^/HLA-DR^-^ while both classical and inflammatory monocytes are all HLA-DR^+^ (51). Similarly, G-MDSC are physically similar to other granulocytes, lineage positive for granulocytes (CD15^+^, CD33^int^, CD66b^+^) but also express the same immunosuppressive markers listed above (51). Meanwhile, i-MDSC are lineage negative (CD14^-^, CD15^-^, CD66b^-^) but maintain expression of CD33 and the immunosuppressive markers (51). While specific markers of the MDSC populations are still being resolved, the gold standard in defining MDSC populations is their functional ability to restructure T cell responses, suppressing classical T cell activation and promoting Treg development.

### MDSCs in GVHD prevention and treatment

MDSCs have been best characterized in the context of solid cancers wherein they have an important role in establishing the immunosuppressive tumor microenvironment (TME); however, they have also been shown to prevent GVHD (52–58). One study found elevated levels of MDSCs in the peripheral blood of patients receiving extracorporeal photopheresis for GVHD treatment relative to both healthy controls and allo-HCT patients without GVHD (55). Another study investigating how post-transplant cyclophosphamide (PTCy) suppresses GVHD identified an integral role for MDSCs in a murine model. In this study, both M-MDSCs and G-MDSCs were elevated in PTCy treated mice from their GVHD model system and were similarly elevated in a cohort of primary allo-HCT patients (54). It has also been shown that umbilical cord blood is rich in MDSCs which may promote the low GVHD rates seen in cord blood transplants (56). Interestingly, the majority of studies that have found elevated MDSC frequencies have also found a similar increase in Tregs levels. The connection between MDSCs and Tregs has been observed in both murine and clinical studies with MDSC depletion studies also showing a subsequent decrease in Treg populations (52, 53, 57). These studies and more have firmly established the hierarchy and importance of MDSCs in promoting the activity of Tregs to suppress GVHD.

### Mechanism driving MDSCs-mediated Treg activity

MDSCs populations have been shown to use a variety of molecular pathways to control T cell responses including indoleamine-pyrrole 2,3-dioxygenase (IDO), cyclooxygenases, arginase, TGF-β, IL-10 and PD-L1 (**Figure 1**). The enzyme IDO catalyzes the rate-limiting step of tryptophan metabolism to L-kynurenine which has been shown to interact with the aryl hydrocarbon receptor (AHR) to drive Treg development and prevent GVHD (58–61). Furthermore, L-kynurenine can inhibit mTOR activation through the activation of GCN2, further driving Treg development (62, 63). The expression of arginase, which degrades arginine, represents another pathway used by MDSCs to dampen immune responses (64–66). The amino acid arginine is an essential amino acid for T cell activation and proliferation. The expression of arginase is induced by IL-10 and TGF-β signaling but is restricted to G-MDSCs in humans, possibly implicating a positive feedback loop between IL-10/TGF-β producing M-DSCS and the development of G-MDSCs (64–66).

Both TGF-β and IL-10 are two well established immunosuppressive cytokines secreted by MDSCs that have a dramatic impact on Treg biology (**Figure 1**). TGF-β is directly connected with the expression of FOXP3 and is an essential component of the polarization of Tregs (67–69). IL-10 meanwhile, has not been shown to be directly involved in the polarization of Tregs but has well established effects on the activation and proliferation of Tregs with the blocking of IL-10 in a mouse model of rheumatoid arthritis sufficient to prevent the immunosuppressive impact of Tregs (70). MDSCs are also potent producers of prostaglandins which have been shown to have both pro- and anti-inflammatory properties. In the context of T cell biology though, recognition of PGE_2_ by the receptors EP2 or EP4 have been shown to prevent the polarization of T cells into TH_1_ cells and directly promote the expression of FOXP3 similar to TGF-β (67, 71–73). Additionally, the prostaglandin PGI_2_ has also been shown to license Treg suppressive activity through the inhibition of the β-catenin pathway (72).

### Factors promoting MDSC development

Since MDSC were first thought to only be immature cell populations derived from aberrant myelopoiesis and granulopoiesis, the biological factors that influence MDSC formation from mature cells have not been as rigorously studied (74). From the limited research into this important biological phenomenon though, the field has begun to coalesce around the hypothesis that MDSCs develop in response to the uptake of extracellular vesicles (which include exosomes) secreted from cells instead of a specific soluble factor or cell contact interaction. To date, studies have found that extracellular vesicles from a variety of cells including melanoma, chronic lymphocytic leukemia and multiple myeloma cells are able to promote MDSC development (48, 75–77). MDSC development from exosomes has also been shown for non-malignant mesenchymal stromal cell (MSC) exosomes (69, 78–81). With the growing evidence that exosomes are sufficient to induce MDSCs, the biological factors controlling this development and the utility of using exosomes as a novel therapy must be explored.

## Exosomes as the next immunosuppressive therapy

Exosomes are a subtype of small lipid bound secreted extracellular vesicles of endosomal origin ranging from 30-150nm that contain and transfer functional cargo consisting of proteins, lipids, microRNAs (miRNAs), mRNA and possibly even DNA (78, 82, 83). Exosomes mediate their biological function through the presentation of proteins/lipids on their surface or by releasing their cargo after internalization. The targeting of exosomes to cell populations is still poorly understood but is thought to be correlated with their interaction with cell surface markers and/or cells possessing phagocytic capacity (e.g., monocytes and macrophages) (84) (**Figure 1**). Exosomes can be produced from a variety of cell populations including Tregs but in this review, we will primarily focus on exosomes derived from mesenchymal stromal cells (MSCs) due to their connection with MDSC development and subsequent expansion of Tregs.

### miRNA driving MDSC development

As an ever-growing number of studies support the role of exosomes in generating MDSC populations, the mechanism(s) utilized by exosomes to generate these MDSC populations remains largely unexplored (**Figure 1**). A focus of several recent studies has been the composition of microRNA (miRNA) cargo within exosomes. miRNAs are short 19-22 nucleotides long and are the most abundant cargo within the exosomes (85). The primary role of miRNA is gene regulation, specifically as post-transcriptional regulation by targeting complementary mRNA and inhibiting protein synthesis (85). While the vast majority of different miRNAs expressed within humans makes this endeavor challenging, several studies have been able to use the miRNA repertoire within exosomes to distinguish healthy from malignant patients (48, 75, 76). One study identified miRNA-10a within exosomes as a driver of multiple myeloma progression while other studies identified miRNA-155 as a driver of MDSCs from melanoma- and CLL-derived exosomes (75, 76, 86). With exosomes often loaded with a multitude of miRNAs and miRNAs able to influence both transcription and post-transcriptional protein expression, studying MDSC development represents an arduous endeavor that is nevertheless essential to fully understand MDSC development (87, 88).

### The use of exosomes in the clinic

Compared to standard cellular therapies, exosomes have several fundamental advantages including i) long-term storage, ii) low-immunogenicity, iii) no requirement for HLA-matching and iv) the ability to be mass-produced (79, 81, 89). Exosome-based therapies also have an advantage over single-agent drugs/therapeutics because they utilize multiple mechanisms to mediate their effect, albeit their exact mechanisms are still being explored (79, 81). At the time of publication, there are currently no FDA approved exosome-based therapies for the treatment of GVHD.

Despite the lack of approved exosome-based therapies, there are a growing number of pre-clinical studies using exosomes to treat a variety of diseases. Most of these studies have utilized MSC-based exosomes, which builds upon the growing field of MSC-based cellular therapies, as they contain similar immunosuppressive properties. A series of studies by one team of investigators have sequentially shown that the treatment with human MSCs, or exosomes “educated” myeloid cells were able to prevent lethal acute radiation syndrome (ARS) (78, 79, 82, 90). In addition, MDSCs generated after treatment with MSC-exosomes were better at promoting spinal injury recovery and revert experimental pulmonary fibrosis (91, 92). In the context of GVHD, several studies have shown that direct human MSCs or MSC-exosome products can ameliorate GVHD when used prophylactically (81, 93–96).

The use of MSCs for GVHD prevention and treatment has been explored in a large number of trials with most reporting that MSCs are safe and well tolerated. Unfortunately, the response rates among trials varied considerably and FDA approval is still elusive (97). Recently, there was one case report on the use of MSC-exosomes for the treatment of chronic GVHD and another for steroid-resistant acute GVHD (81, 98). While these groups reported that MSC-exosomes were well tolerated with an overall beneficial impact on the patient, larger multi-center trials will be needed to determine if MSC-exosomes therapies can succeed where MSC cell therapies could not. With the ability to generate exosomes in large quantities, their ability to be frozen and used “off the shelf” for multiple injections and their ability to be used in the allogeneic setting without a loss in functional capacity, all indicate that exosome-based therapies are poised to grow tremendously in the next decade.

## Conclusion

The field of GVHD research is at an exciting inflection point where the investigation into biologics as novel prophylaxis/treatment modalities may soon become standard. While the three modalities discussed in this review are all poised to make significant advances toward clinical use, these biologic modalities are not without their limitations. Similar to the experiences of CAR manufacturing, the use of Tregs or MDSCs as a cellular therapy will always be challenged with their production times, the manufacturing process and capacity to be used in the allogeneic setting. For these reasons, MSC-exosome treatment represents an exciting and promising modality. Exosome treatments have the potential to be used in the allogeneic setting, mass-produced for repeated injections, and stored for immediate use. Their eventual use in the clinic will ultimately be decided by their ease of use and long-term efficacy compared to direct cellular therapies which boast strong short term activity but are limited to a single infusion and long-term activity.

Promoting the expansion and activation of Tregs after allo-HCT remains the most validated method of suppressing GVHD though the optimal approach to achieve this remains under investigation. Exploring the use of MDSCs and MSC-exosomes as novel approaches to activate Tregs should be a focus of investigation in the next decade. The development of these and other biologic-based prophylaxis/treatment modalities will offer allo-HCT recipients an improved quality of life by reducing off-target toxicity and improving overall efficacy by directing their new immune system to control the development of GVHD.

## Author contributions

NH drafted and performed the literature search. NH, JK and PH revised and edited the manuscript. All authors approved the final version of this manuscript. All authors contributed to the article and approved the submitted version.
